# The Integration of Traditional Medicine with Conventional Biomedicine: A Narrative Review of the Japanese Perspective

**DOI:** 10.1089/jicm.2022.0643

**Published:** 2023-06-06

**Authors:** Tetsuhiro Yoshino, Akihiko Kashio, Yoshihiro Terasawa, Marina Hachiki, Ryo Yoshinaga, Ryutaro Arita

**Affiliations:** ^1^ Center for Kampo Medicine, Keio University School of Medicine, Shinjuku-ku, Japan.; ^2^ Kyuden Family Clinic, Setagaya-ku, Japan.; ^3^ Department of General Internal Medicine, Kuchinotsu Hospital, Minamishimabara, Japan.; ^4^ General Medicine, Dokkyo University Hospital, Shimotsuga-gun, Japan.; ^5^ Department of Japanese Oriental (Kampo) Medicine, Iizuka Hospital, Iizuka-shi, Japan.; ^6^ Department of General Internal Medicine, Kyushu University Hospital, Fukuoka, Japan.; ^7^ Department of Kampo and Integrative Medicine, Tohoku University Graduate School of Medicine, Sendai, Japan.; ^8^ Department of Education and Support for Regional Medicine, Department of Kampo Medicine, Tohoku University Hospital, Sendai, Japan.

**Keywords:** aging society, Kampo medicine, universal health insurance coverage, free health care access

## Abstract

**Objectives:**

This is a narrative review of the integration of traditional medicine with conventional biomedicine in present day Japan, whose aging population is considered one of the largest globally.

**Design:**

It is focused on the aging population because this age group most avails of healthcare. We also tried to describe the unique Japanese medical situations, clinical outcome of Japanese traditional medicine (Kampo medicine) which may include acupuncture, and education of Kampo medicine workforce.

**Results:**

Conventional schools of medicine in Japan are required to teach Kampo medicine, and most Japanese physicians (>80%) prescribe traditional medicine, especially in primary care settings. The universal national healthcare system covers Kampo medicine prescribed by physicians and treatment by acupuncturists (they sometimes refer patients who may need evaluation by physicians), enhancing access to primary healthcare. Additionally, pharmacists who graduated from conventional schools of pharmacy also select and sell Kampo medicine as over-the-counter (OTC) medication. Kampo medicine available as prescription drugs and OTC is effective, and has been proven to be economically beneficial in several clinical settings.

**Conclusions:**

An aging population is a global concern for both developed and developing countries. Japan, having a significantly-large aging population, integrates conventional biomedicine and traditional medicine in its universal national healthcare coverage, through its biomedically-trained physicians and pharmacists who also learned traditional medicine, as well as the acupuncturists. By reviewing the current situation in Japan, the authors hope to introduce the future of the global contribution of traditional, complementary, and integrative medicine in primary care.

## Introduction

The 2018 Declaration of Astana from the World Health Organization (WHO) and the United Nations Children's Fund is a landmark step forward for primary health care; public health; and traditional, complementary, and integrative medicine. The WHO's 1978 Alma-Ata Declaration prioritized the international importance of Universal Health Coverage. The Declaration is the world's first primary health care document that explicitly recognizes the value and importance of traditional health care systems in the successful delivery of primary health services. However, these declarations are considered just for developing countries and discussed in the context of international development assistants in Japan, and are almost ignored in the Japanese domestic medical system.

The term primary health care constitutes public health aspects. Public health, including water supply, food security, and infection control in Japan, is practiced by health care practitioners who graduated from nursing school and are supervised by public health-specializing physicians. For example, the water supply penetration rate in Japan is 100% in urban areas and 98%–99% nationwide.^1^ Such a lifeline is well within reach and usually regarded as a government and municipal issue. In contrast, primary care providers emphasize personal, familial, and community health nationwide. Although Japan is one of the most developed countries in the world, health disparities exist; however, primary care is provided for everyone in Japan regardless of their economic, racial, religious, and educational status. The traditional medicine system integrated with conventional biomedicine should play an important role in Japan. Our health system could be a role model for any country despite their national developing status to improve primary health care for each individual. Therefore, this review is focused on improving health care on an individual basis in Japan.

In public medical health care in Japan, known for its aging population, conventional biomedicine plays a central role. The national health care system covers a part of complementary medicine as summarized in Table 1 and is integrated with conventional biomedicine. The integrative approach is used in primary care, especially for elderly patients with multimorbidity.^2,3^ However, the health care systems of coverage, reimbursement, license, and education are complicated. The clinical effectiveness based on research has not been adequately revealed because traditional and complementary medicine have been established based on empiric therapy. In this review, we demonstrated the current situation of traditional and complementary medicine in a modern society in Japan.

**Table 1.** Classification of Complementary and Alternative Medicine Modalities in This Review Covered by the National Health Insurance in Japan

	Kampo formula	Topical NSAIDs	Oral vitamins	Acupuncture/Moxibustion	Massage/Amma/Shiatsu	Judo therapy
Indications covered by health care insurance	148 Medications all have their own indications	>30 Brands all have their own indications	>50 Brands all have their own indications	6 Ailments	Muscle atrophy and muscle contracture	Traumatic injury
Restrictions	*If* prescribed by physicians or dentists	*If* prescribed by physicians	*If* prescribed by physicians	*If* agreed upon by physicians	*If* agreed upon by physicians	*If* agreed upon by physicians
Uncovered	OTC drugs	OTC drugs	OTC drugs	Possible	Possible	Possible
National License	Physician Dentist Pharmacist	Physician Pharmacist	Physician Pharmacist	Acupuncturist Moxibustionist Physician	Anma, shiatsu, and massage therapist	judo therapist

NSAID, nonsteroidal anti-inflammatory drug; OTC, over-the-counter.

## Overview of Japan's Current Aging Population

Japan's total population in 2020 was 125.71 million, ranking 11th in the world according to the Statistical Handbook of Japan 2021 by the Statistics Bureau, Japan.^4^

In Japan, the aging population (65 years old and over) exceeded 10% in 1985. However, as of 1950, it was already 11.4% in France and 10.2% in Sweden. The percentage exceeded 10% in 1955 in Germany, 1965 in Italy, and 1970 in the United States. Recently, the aging population in Japan increased quite rapidly as compared with the United States and European countries; its aging population constituted 26.6% of the total population in 2015, far exceeding the ratio in the United States (14.6%), France (18.9%), Sweden (19.6%), Germany (21.2%), and Italy (21.9%), in the same year. The ratio of Japan's aging population increased to 28.8% in 2020, the highest in the world, and this number is estimated to increase more until the 2040s.^4^

Notably, income inequality leads to health inequality.^5^ Income inequality among households with elderly members decreased overall after the mid-1980s due to the decline in three-generation households,^6^ meaning, many aging people become “equally” poor living without their working-aged children. Furthermore, nonregular employment among young and middle-aged people is rapidly spreading in recent years in addition to an increase in the unemployed aging population.^7^ The poverty rate in Japan is lower than that in the United States and South Korea, almost the same as that in Spain or Italy, higher than that in Canada or Germany, and twice as high as that in France or the Netherlands.^8^ In addition to income inequality, regional and educational disparities have also been reported to be associated with health disparities in Japan.^9–11^

## The Universal Health Insurance Coverage

The universal health insurance coverage system started in 1961 in Japan. Unlike United Kingdom where there is one insurer by taxation, there are several insurers in Japan. They are national, municipal, or employment based, and all follow the same medical payment system and coinsurance rule.^12,13^ Coinsurance means a payment required of an insured person for that portion of medical expenses and could be reduced according to their older age and lower income. If it is covered by health insurance, even the most advanced and expensive medical treatment can be received regardless of income or insurance payment. There is no difference in insurance-covered treatment among the insurers. Since the government stands for mutual aid (medical insurance) for large risks and self-help (each individual) for small risks, the priority is to guarantee the availability of expensive new drugs using social security funds.

Traditional Japanese medicines (Kampo formulas), as well as vitamins, and topical nonsteroidal anti-inflammatory drugs (NSAIDs) prescribed by physicians, are covered by health insurance (Table 1) from early days. The importance of Kampo medicine was recognized at that time, and Kampo medicine was included in the national health insurance system and gradually accepted in the Japanese medical system (as per political policies). The decision to cover Kampo formulas in health insurance is not based on clinical trials, and clinical indications for each formula are based on the practices and disease classifications at that time. The first Kampo formulas were covered by insurance in 1967, and currently, 147 intake Kampo formulas and 1 topical Kampo ointment are covered in Japan.

Most physicians (more than 80% of the respondent physicians in Japan, especially primary care physicians) prescribe Kampo formulas in their daily clinical practice.^14–18^ Kampo formulas are the most common modality of Complementary and Alternative Medicine (CAM) recommended by physicians in Japan.^19^ The market size of prescribed Kampo formulas has been increasing to 162 billion yen/year in 2020.^20^

Additionally, Kampo formulas are popular as over-the-counter (OTC) medications that people keep at home,^19,21^ even though the dosage is sometimes lower than prescription medicines. Pharmacists select the OTC Kampo formula and play an important role in preclinic-level self-care, such as treatment of the common cold or allergic rhinitis. Historically, pharmacists passed the Kampo tradition for about a century from the proliferation of Kampo specialists in the mid-late 19th century to the mid-20th century. This is one reason why OTC Kampo is widely accepted in the Japanese medical system. The market size of the OTC Kampo formulas is about 50 billion yen/year in 2020, about the same as that of antipyretic analgesics and topical NSAIDs.^20^

Furthermore, acupuncture, moxibustion, massage, anma, shiatsu, and Judo therapy by specialist practitioners can also be covered by health insurance in certain situations. Yoga, meditation, music therapy, and other CAM modalities are not covered by the health insurance system in Japan.

Moxibustion is a type of heat therapy in which mugwort named moxa is burned on or above the skin to warm and stimulate an acupoint or affected area. Anma and shiatsu are massage therapies performed over the clothes where anma practitioners basically rub from proximal to distal in contrast to massage therapists' rub from distal to proximal, and shiatsu therapists just press the acupoints. Judo therapy is a manual therapy for bone, muscle, and joint injuries. It is paid for through medical care reimbursement (cash payment) by the insurer to the patients, usually several months after the treatment if the application for reimbursement is approved; meanwhile, the coinsurance is charged at the time of treatment. Additionally, the diseases covered are limited. There are only six ailments covered by health insurance for acupuncture and moxibustion, and these include low back pain and neuralgia.

Muscle atrophy and muscle contracture are treated with anma and massage, while several ailments such as an acute fracture or chronic pain after trauma are alleviated by judo therapy.

Moreover, such treatment options cannot be used in combination with conventional biomedicine or the Kampo formulas prescribed by physicians. Only a physician or a CAM-specialist practitioner can be reimbursed per one ICD-10 code in 1 month, and the physician controls whether the practitioner can be reimbursed for the given condition. Practitioners must have a written consent form from physicians for health insurance coverage on acupuncture, moxibustion, anma, shiatsu, massage, and judo therapy, but some orthopedic physicians hesitate to give up their reimbursement rights for a patient and give their consent, especially for chronic pain. Therefore, if physicians claim medical expenditure for the same disease in the same month, the patients may not be reimbursed for their treatment by the CAM practitioners even with the consent form from the physicians. This is one of the hurdles in integrating the various CAM practices other than Kampo formulas and topical NSAIDs prescribed by physicians into the national health insurance system in Japan.

Social security costs, including medical costs, are increasing annually mainly due to the rapid population aging and the recent introduction of expensive biomedicine.^13^ In addition, the decline in tax revenues due to the decrease in the working population and the increase in medical costs have become major social problems.^22^ The financial situation of each insurer differs considerably, and due to the imbalance in population composition, the financial management of national health insurance was changed from smaller municipal to larger prefectural units in 2018. This is also the case for employment-based insurers. Several larger companies have stopped providing health insurance for their employees individually, and insurers have been merged into a public-cooperation-run health insurer.

There has been some discussion about excluding Kampo formulas, vitamins, and topical NSAIDs from insurance coverage,^23,24^ but this has yet to be realized due to the patients' strong desire for continued insurance coverage. It is less expensive to use the Kampo formulas in several situations such as flu,^25^ chronic subdural hematoma,^26^ and postoperative adhesive small bowel obstruction.^27^

## Free Access

Anyone can avail of medical services in a medical institution at any time through health insurance regardless of their income. Moreover, patients can visit not only local primary care physicians but also advanced medical institutions such as university hospitals, at request. Ambulances can also be used free of charge by anyone, including travelers without Japanese health insurance.

Many Japanese patients hesitate to make medical treatment and care decisions for themselves without consulting family, close friends, or someone considered superior, especially physicians.^28^ Thus, Japanese are highly dependent on medical care and have little awareness of autonomous health maintenance. This might be because the out-of-pocket cost is kept uniformly low throughout the country. In general, the out-of-pocket costs for the first physician consultation and the revisit cost several U.S. dollars. Lower out-of-pocket cost enables patients to visit health care institutions frequently, which results in polypharmacy.^29^ Kampo formulas are composed of several crude drugs that enable physicians to treat several symptoms with one formula, leading to one solution.

Officially, a person does not have a primary health care physician. Because insurers impose no restrictions, patients may voluntarily continue to visit their nearby general practitioner, but children and working-age adults, in particular, tend to have less contact with primary care. Additionally, low-income individuals in Japan have poorer access to outpatient care and more serious health conditions than their higher-income counterparts.^30^ The take-up rate of annual health checkups is also low even though patients can receive health checkups free of charge at the insurer's expense. Conversely, the elderly often have monthly primary care visits with much cheaper coinsurance and out-of-pocket costs than younger people.

The distinction between primary care and secondary/tertiary care is unclear in Japan.^22^ Patients can directly visit more-expensive medical institutions at their discretion; since 2015, additional charges on out-of-pocket costs have been imposed for visits to large hospitals without a referral letter from primary care physicians. Outpatient clinics specializing in Kampo medicine exist, but often patients come to these clinics by their own choice rather than through a referral by their physicians.^31,32^ The examination records are often not shared with patients when they visit a higher-level medical facility, resulting in repeated and unnecessary examinations since medical records are considered the property of the medical facility and patients cannot handle such records themselves. Also, this makes it difficult for CAM practitioners to collect medical information on their new patients.

Kampo formulas were used in 22.8% of symptomatic patients, with no significant regional differences, whereas physical therapy such as acupuncture and moxibustion was used in 7.4% of symptomatic patients, and was more common in urban areas according to a national survey,^33^ possibly due to the imbalanced distribution of specialists for such treatments. One study performed in an urban hospital reported that there was no significant difference in CAM use for the different age groups, education levels, and financial status,^34^ whereas another study, performed in a rural clinic, reports that female respondents, individuals with higher levels of education, and those with poorer overall health status, were more likely to use CAM.^35^

Many people, especially the elderly and women in Japan, consult primary care physicians regarding their undiagnosed complaints after having no abnormalities in their conventional biomedical tests (often referred to as illness); they are requesting a physician's guidance in the use of CAM.^36^ Authors believe that selecting a Kampo formula for the patient is not only applying an alternative herbal medicine to the given symptom but physicians need to take a holistic approach considering both physiological and psychological health together. Primary care physicians take a biopsychosocial approach, considering the psychosocial aspects aside from the biomedical content (often referred to as disease) that are mainly studied in a medical school.^17,18^ Similarly, Kampo medicine is based on a whole-person approach that considers both physiological and psychological health together. The basic concept of primary care and Kampo medicine is patient-centered medicine, emphasizing the integrated understanding of the disease, illness, and health that enhances the patient–clinician relationship.

Additionally, primary care physicians undertaking the whole-person approach of traditional Kampo medicine also understand patients' complaints based on the empirical Kampo concept, which may enhance patients' understanding of their illnesses. This leads patients to strengthen their self-efficacy and supports them in self-healing. Therefore, visiting a Kampo outpatient clinic and a primary care outpatient clinic may provide similar experience for patients. Kampo physicians are expected not only to be specialists in Kampo medicine and CAM but also to be gatekeepers, providing preventive medicine and, if needed, giving an appropriate Western medical diagnosis, and connecting patients to specialists as needed.

## Clinical Outcome

Kampo formulas are included in guidelines for diseases common in the primary care field, such as hypertension, back pain, and chronic pain.^37–39^ Some hospitals include Kampo formulas in their coordinated care plans (clinical pathways) along with conventional biomedicine, especially after open laparotomy. Whereas, acupuncture finds limited mention in the guidelines, and even that it is often accompanied by a negative viewpoint or misunderstanding of the clinical evidence.^40,41^

Kampo formulas have been included in the universal health insurance system with conditions since the 1960s or 1970s. However, a discrepancy exists between the current actual use of the Kampo formulas and its insured indications because of insufficient, or even the absence of, clinical trials performed for obtaining the indications of Kampo formulas. Therefore, phase 3 clinical trials have been performed, sponsored by pharmaceutical companies or national research funding even after obtaining its insured indications.

The Japan Society for Oriental Medicine (JSOM) prepared Evidence Reports of Kampo Treatment (EKAT), and the latest clinical studies, including randomized controlled trials, are listed on the JSOM webpage.^24,42^

Similarly, the Ministry of Health, Labor, and Welfare and the Japan Agency for Medical Development are actively promoting the accumulation of scientific evidence reports for the use of integrative medicine. Similar evidence reports for various CAM modalities, not limited to Kampo, is being gathered by evidence-based Japanese integrative medicine. There is a collection of articles on the webpage evidence-based Japanese Integrative Medicine (eJIM) on such topics as acupuncture and moxibustion;^43,44^ anma, massage, and shiatsu and yoga.^45^

Acupuncture is rarely practiced in hospitals, even in teaching hospitals, due to the abovementioned high reimbursement hurdles, but some university hospitals, such as the Tohoku, Hiroshima, Saitama, Tokai, and Keio University hospitals have acupuncturists in their faculty.^17^ These hospitals gave up providing acupuncture under health insurance coverage partially due to a need to treat complicated patients with diseases other than the six ailments covered by the insurance.

As mentioned above, most physicians in Japan prescribe Kampo formulas in their daily clinical practice, although there is still room to improve the selection of Kampo formulas.^16^ In several situations, prescriptions are not necessarily based on traditional diagnoses patterns, but are merely prescribed as a proxy for general Western biomedicine for medical illnesses and symptoms.

**FIG. 1.**
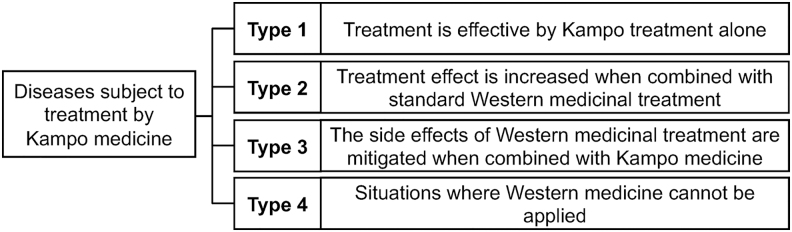
The clinical situations in which Kampo formulas are used classified by Yasui. Type 1: Symptom improvement can be expected with Kampo formula alone; Type 2: Conventional biomedicine effects are enhanced with Kampo formula; Type 3: Biomedicine side effects are alleviated by Kampo formula; and Type 4: Kampo formula is administered as an alternative when biomedicine is not available due to drug allergy or that it is too expensive for the patient.

The clinical situations in which the Kampo formula is used are classified into four categories as proposed by Yasui (Fig. 1). Type 1: Symptom improvement can be expected with Kampo formula alone; Type 2: Conventional biomedicine effects are enhanced with Kampo formula; Type 3: Biomedicine side effects are alleviated by Kampo formula; and Type 4: Kampo formula is administered as an alternative when biomedicine is not available due to drug allergy or is too expensive for the patient.^46^

## Education and the Workforce

In China and Korea, one can become a Chinese or Korean doctor by graduating from a university specializing in Chinese or Korean medicine, but in Japan, there is no such qualification and one must graduate from a medical school specializing in conventional biomedicine.^47^ No universities have been established to specialize in teaching Kampo medicine. The Japanese medical licensing system's refusal to recognize Kampo doctors dates back to the establishment of the medical licensing system in the Meiji era in the late 19th century. This decision, however, opened Japan's integrative medicine health care system.

Since 2002, despite insufficient time, education on Kampo medicine is provided in all medical and pharmacy schools. In addition, few medical school faculty members can teach Kampo, and it is difficult to train them in a short period.^48,49^ It is important to be able to provide standardized education in Kampo medicine even where there are no experts. The Japan Council for Kampo Medical Education (JCKME) is an organization of Kampo educators from 82 medical schools in Japan, including primary care physicians, “Lectures on Kampo Medicine” has been published as a standard textbook, and standard slides have been prepared by JCKME. Additionally, many students who want to learn primary care also want to learn Kampo medicine, and there is a private online students' group for learning Kampo medicine and primary care medicine simultaneously. The use of Kampo medicine will become more widespread when the generation of primary care physicians who had pregraduate education in Kampo medicine become attending physicians.

Acupuncturists, moxibustion specialists, anma, massage, shiatsu therapists, and judo therapists, are nationally certified with a minimum of 3 years of vocational school education based on the “Act on Practitioners of Massage, Finger Pressure, Acupuncture, and Moxacauterization, etc.” Japan College Association of Oriental Medicine publishes a Standard Textbook of General Clinical Medicine for students at the colleges. It is also possible to practice acupuncture and moxibustion for physicians in Japan. In contrast, chiropractic practice and osteopathy, widely used in the United States, and any other treatment modalities such as Homeopathy and Ayurveda are not authorized by the Japanese government.

Fewer than 2000 Kampo specialists are trained exclusively through postgraduate education among ∼300,000 physicians in Japan. While anyone with a Japanese medical license can prescribe Kampo, a board-certified Kampo specialist by JSOM is regarded as a subspecialty and the applicant is required to possess board certification in conventional biomedicine before starting the 3-year residency program. Residents in the residency program have to attend clinics, treat patients on their own choice of Kampo formulas, and write 10 case reports. After the 3-year residency program, candidates take a final examination that covers not only Kampo formulas but also acupuncture. This condition is one aspect of integrative medicine in Japan.

Postgraduate clinical training is provided only in conventional biomedicine, which limits the environment for learning about Kampo medicine, and interest in Kampo may wane during this time for some residents. In addition, postgraduate education for physicians interested in Kampo medicine is lacking, although they do not want to obtain board certification in Kampo medicine. Similar to how all diabetes patients cannot be treated by diabetes specialists, the involvement of Kampo nonspecialist physicians, especially primary care physicians in Kampo medicine, is essential to achieving efficient and effective Kampo treatment.

The standard textbook “Lectures on Kampo Medicine” by JCKME, lectures by JSOM, and manufacturer-led postgraduate education are common for such physicians. Indeed, Japan's primary care association holds sessions on Kampo medicine supported by JSOM during its annual conference and many primary care physicians attend these sessions. As primary care physicians who do not specialize in Kampo medicine have more opportunities to use them, their next challenge will be to use Kampo medicines appropriately and safely.

Developments in technology-aided decision support systems will help primary care physicians who do not specialize in Kampo medicine by programming prescription choices of Kampo formulas. Kampo nonspecialist primary care physicians can refer difficult-to-treat patients with their “first-choice” Kampo formulas to Kampo specialists and Kampo specialists reverse refer the stable patients to primary care physicians.

Physicians are free to choose their specialty and region of practice without any restrictions.^50^ There is no renewal system for medical licenses, and continuous medical education is not mandatory. The Japan Medical Association states that “physicians should be motivated to pursue a lifelong education at their own initiative,”^51^ and this policy is widely accepted in Japan. Furthermore, when starting their practice after working at a teaching hospital, they can freely set their specialty (other than anesthesiology and dentistry), so that even if they have worked in a surgical specialty at a teaching hospital, they can work as a primary care physician afterward. This career path is quite major, as a private primary care physician often earns more than a hospitalist.^52^ Many physicians who have not worked close to primary care notice the importance of the biopsychosocial approach when they open their private practices. We believe it is important to include Kampo medicine as a treatment option in the outpatient setting to help cultivate the biopsychosocial and whole-person approach even in a specialty care setting at teaching hospitals.

There had been no official specialty for primary care, and the specialty was finally established in Japan in 2018, with the start of the training for general practitioners in the same year that emphasizes patient-centered care and requires training in Kampo medicine. The field of family physicians in outpatient clinics and general practitioners in hospitals is expanding in Japan.

Since physicians' choice of specialty and region of practice is not restricted, there are serious shortages in several specialties relating to primary care such as emergency medicine, pediatrics, and obstetrics. There are two aspects to the shortage: the shortage of physicians per population and per area. There is a serious shortage of physicians per specified population in urban areas, especially around the Tokyo district,^53^ and a serious shortage per area in depopulated places such as Hokkaido and the Tohoku district.^54^

During the coronavirus disease 2019 (COVID-19) pandemic, there was a serious shortage of manpower to deal with febrile patients in the emergency room.^55^ Kampo specialists at Tohoku University, who also routinely provide primary care, achieved good results in the treatment of patients suffering from COVID-19.^56^ At Tohoku University, the Department of Kampo Medicine is one of the divisions in the Department of Education and Support for Regional Medicine that play a role in primary care in rural areas and the disaster medicine field.^57–59^ In addition, infant deliveries are not handled by primary care physicians in Japan and are instead handled by obstetricians or midwives.

However, Kampo medicine is often used to treat various ailments of pregnant or postpartum women.^60^ Moxibustion, which involves the burning of the herb moxa over acupuncture points has been found to reduce the number of noncephalic presentations at term.^61^

## Conclusions

An aging population is a global concern for developed as well as developing countries and it increases the importance of primary care.^31^ Japan has five decades of experience in integrative medicine that is at least partially covered by the universal national health insurance coverage and patients can enjoy free access to medical institutions according to their preference. By reviewing the current situation in Japan, the authors hope to introduce the future of the global contribution of traditional, complementary, and integrative medicine in primary care.

The simple tasks of selecting and dispensing prescriptions according to guidelines will be taken out of human hands as a result of technological developments. In this way, the work of physicians and pharmacists in primary care will place greater emphasis than ever on communication with patients and collaboration among multidisciplinary professions, and will require a holistic view of the patient. From one perspective, the role of CAM, including Kampo medicine, which has traditionally provided whole-person treatment, will be reevaluated, and found needed more than ever before.
